# Supplementary material for: Health-related quality of life and associated factors among cancer patients in Ethiopia: Systematic review and meta-analysis
Source: PLoS One. 2022 Dec 1;17(12):e0277839. doi: 10.1371/journal.pone.0277839 (PMC9714884; doi:10.1371/journal.pone.0277839)
Supplement: S1 File — (DOCX) [file pone.0277839.s001.docx]

**Identification of studies via databases and registers**

Records removed *before screening*:

Duplicate records removed (n =201)

Records marked as ineligible by automation tools (n = 3125)

Records removed for other reasons (n = 253)

Records identified from*:

Databases (n =3583)

Registers (n =83)

**Identification**

Records screened

(n = 340)

Records excluded**

(n =135)

Reports sought for retrieval

(n = 205)

Reports not retrieved

(n =118)

**Screening**

Reports assessed for eligibility

(n = 87)

Reports excluded:

Reason 1 (n =21)

Reason 2 (n = 27)

Reason 3 (n =8)

etc.

Studies included in review

(n = 19)

Reports of included studies

(n =12)

**Included**

**Figure 1.** PRISMA diagram of selecting and including studies for a systematic review and meta-analysis for the prevalence of health-related quality of life of cancer patients in Ethiopia, 2021.

*Consider, if feasible to do so, reporting the number of records identified from each database or register searched (rather than the total number across all databases/registers).

**If automation tools were used, indicate how many records were excluded by a human and how many were excluded by automation tools.

*From:*  Page MJ, McKenzie JE, Bossuyt PM, Boutron I, Hoffmann TC, Mulrow CD, et al. The PRISMA 2020 statement: an updated guideline for reporting systematic reviews. BMJ 2021; 372:n71. doi: 10.1136/bmj.n71

For more information, visit: <http://www.prisma-statement.org/>
